# Supplementary material for: Transcriptional correlates of malaria in RTS,S/AS01-vaccinated African children: a matched case–control study
Source: eLife. 2022 Jan 21;11:e70393. doi: 10.7554/eLife.70393 (PMC8782572; doi:10.7554/eLife.70393)
Supplement: Supplementary file 2. [file elife-70393-supp2.docx]

**Supplementary Table 3.** **Numbers, age group, and case-control status of RTS,S/AS01 recipients by site for whom month 0 and/or month 3 PBMC samples were included in the ICS/immunophenotyping analysis.**

| **Month 0 (Overall Total 73)** | | | | | | | | | | | |
| --- | --- | --- | --- | --- | --- | --- | --- | --- | --- | --- | --- |
| Cases (n = 17) | | | | | | Controls (n = 56) | | | | | |
| Bagomoyo | | Manhiça | | Lambaréné | | Bagomoyo | | Manhiça | | Lambaréné | |
| Infants | Children | Infants | Children | Infants | Children | Infants | Children | Infants | Children | Infants | Children |
| 0 | 14 | 0 | 1 | 0 | 2 | 0 | 54 | 0 | 0 | 0 | 2 |
| **Month 3 (Overall Total 182)** | | | | | | | | | | | |
| Cases (n = 37) | | | | | | Controls (n = 145) | | | | | |
| Bagomoyo | | Manhiça | | Lambaréné | | Bagomoyo | | Manhiça | | Lambaréné | |
| Infants | Children | Infants | Children | Infants | Children | Infants | Children | Infants | Children | Infants | Children |
| 0 | 12 | 19 | 1 | 3 | 2 | 0 | 51 | 77 | 2 | 10 | 5 |
