# Supplementary material for: Transcriptional correlates of malaria in RTS,S/AS01-vaccinated African children: a matched case–control study
Source: eLife. 2022 Jan 21;11:e70393. doi: 10.7554/eLife.70393 (PMC8782572; doi:10.7554/eLife.70393)
Supplement: Supplementary file 4. — These individual genes were obtained by looking at gene-level correlate heatmaps (Figure 6—figure supplements 2–9) of the eight blood transcriptional modules (BTMs) (M4.0, S10, S5, M168, M4.3, M11.0, M4.15, and S4) that significantly associated with risk in MAL067 and in two controlled human malaria infection (CHMI studies). The table also contains information on whether each individual gene also significantly associated with risk in a CHMI study (check mark; columns 2 through 4). [file elife-70393-supp4.docx]

**Supplementary Table 5. List of 35 individual genes whose baseline expression in vehicle-treated PBMC significantly associated with risk in MAL067.** These individual genes were obtained by looking at gene-level correlate heatmaps (Figure 6-figure supplements 2-9) of the eight BTMs (M4.0, S10, S5, M168, M4.3, M11.0, M4.15, and S4) that significantly associated with risk in MAL067 and in two CHMI studies. The table also contains information on whether each individual gene also significantly associated with risk in a CHMI study (check mark; columns 2 through 4).

| **Gene** | **WRAIR 1032** | **MAL068 RRR** | **MAL071 RRR** |
| --- | --- | --- | --- |
| *CA2* |  |  |  |
| *CALCRL* |  |  |  |
| *CCNB1* |  |  |  |
| *CCNF* |  |  | ✓ |
| *CCR1* | ✓ |  |  |
| *CENPE* |  |  |  |
| *CFD* | ✓ |  |  |
| *CSF2RB* |  |  |  |
| *CTSH* |  |  |  |
| *CYBRD1* |  |  |  |
| *DAPK1* |  |  |  |
| *DMXL2* |  |  |  |
| *GGH* |  |  |  |
| *HNMT* |  |  |  |
| *IL13RA1* | ✓ |  |  |
| *KIF18A* |  | ✓ |  |
| *MAFB* | ✓ |  |  |
| *MK167* |  |  | ✓ |
| *MS4A4A* |  |  |  |
| *MSR1* |  |  |  |
| *NPL* | ✓ |  |  |
| *P2RY6* |  |  |  |
| *RAB32* |  |  |  |
| *RBM47* | ✓ |  |  |
| *REC3* |  |  |  |
| *RIN2* |  |  |  |
| *RNASEH2A* |  |  |  |
| *RXRA* |  |  |  |
| *SEMA4A* |  |  |  |
| *SGK1* |  |  |  |
| *SLC31A2* |  |  |  |
| *STAB1* |  |  |  |
| *TLR4* |  |  |  |
| *TLR5* |  |  |  |
| *TNFRSFIIA* |  |  |  |
