# Supplementary material for: Transcriptional correlates of malaria in RTS,S/AS01-vaccinated African children: a matched case–control study
Source: eLife. 2022 Jan 21;11:e70393. doi: 10.7554/eLife.70393 (PMC8782572; doi:10.7554/eLife.70393)
Supplement: Transparent reporting form [file elife-70393-transrepform1.docx]

***eLife’s* transparent reporting form**

We encourage authors to provide detailed information *within their submission* to facilitate the interpretation and replication of experiments. Authors can upload supporting documentation to indicate the use of appropriate reporting guidelines for health-related research (see [EQUATOR Network](http://www.equator-network.org/%20)), life science research (see the [BioSharing Information Resource](https://biosharing.org/)), or the [ARRIVE guidelines](http://www.plosbiology.org/article/info:doi/10.1371/journal.pbio.1000412) for reporting work involving animal research. Where applicable, authors should refer to any relevant reporting standards documents in this form.

If you have any questions, please consult our Journal Policies and/or contact us: [editorial@elifesciences.org](mailto:editorial@elifesciences.org).

**Sample-size estimation**

- You should state whether an appropriate sample size was computed when the study was being designed
- You should state the statistical method of sample size computation and any required assumptions
- If no explicit power analysis was used, you should describe how you decided what sample (replicate) size (number) to use

Please outline where this information can be found within the submission (e.g., sections or figure legends), or explain why this information doesn’t apply to your submission:

No explicit power analysis was used. We used all available samples from infants and children participating in the immunology study ancillary to the RTS,S phase 3 clinical trial and who had malaria during follow-up (malaria cases). In addition, we selected 2-4 matched non-malaria controls for each malaria case, prioritizing subjects who had samples from both, pre-vaccination and post-vaccination.

This information can be found at the Materials and Methods section, subsection “MAL067 trial”

**Replicates**

- You should report how often each experiment was performed
- You should include a definition of biological versus technical replication
- The data obtained should be provided and sufficient information should be provided to indicate the number of independent biological and/or technical replicates
- If you encountered any outliers, you should describe how these were handled
- Criteria for exclusion/inclusion of data should be clearly stated
- High-throughput sequence data should be uploaded before submission, with a private link for reviewers provided (these are available from both GEO and ArrayExpress)

Please outline where this information can be found within the submission (e.g., sections or figure legends), or explain why this information doesn’t apply to your submission:

Sample libraries that exhibited less than 75,000 total RNAseq reads per sample were of low quality. Thus, such libraries were removed from the study.

Genes that had less than 20 samples (around 10%) with read counts greater than 5 were also removed. This information can be found at the Materials and Methods section, Data processing and statistical analysis (page 10).

For intracellular cytokine staining and flow cytometry data we used two standard criteria to filter poor quality samples:

1. Samples with high background: samples where the non-stimulated sample magnitude is > 10%.

2. Low quality samples where the number of CD4 T cells is less than 20000 cells.

No subjects were flagged as high background and 85 were flagged as having low T cell counts. These were removed from the analysis.

Table 1 shows the number of samples analysed by RNAseq in the study.

Supplementary Table 1 provides additional information on the individuals included in the case control analysis.

Supplementary Table 3 reports the number of samples analysed by flow cytometry.

Code will be available.

**Statistical reporting**

- Statistical analysis methods should be described and justified
- Raw data should be presented in figures whenever informative to do so (typically when N per group is less than 10)
- For each experiment, you should identify the statistical tests used, exact values of N, definitions of center, methods of multiple test correction, and dispersion and precision measures (e.g., mean, median, SD, SEM, confidence intervals; and, for the major substantive results, a measure of effect size (e.g., Pearson's r, Cohen's d)
- Report exact p-values wherever possible alongside the summary statistics and 95% confidence intervals. These should be reported for all key questions and not only when the p-value is less than 0.05.

Please outline where this information can be found within the submission (e.g., sections or figure legends), or explain why this information doesn’t apply to your submission:

Methods section, Data processing and statistical analysis

Exact values of N are in Table 1 and Table S3 and in Figure legends.

(For large datasets, or papers with a very large number of statistical tests, you may upload a single table file with tests, Ns, etc., with reference to sections in the manuscript.)

**Group allocation**

- Indicate how samples were allocated into experimental groups (in the case of clinical studies, please specify allocation to treatment method); if randomization was used, please also state if restricted randomization was applied
- Indicate if masking was used during group allocation, data collection and/or data analysis

Please outline where this information can be found within the submission (e.g., sections or figure legends), or explain why this information doesn’t apply to your submission:

Methods section, “MAL067 trial subsection”, Table 1, Table S3

**Additional data files (“source data”)**

- We encourage you to upload relevant additional data files, such as numerical data that are represented as a graph in a figure, or as a summary table
- Where provided, these should be in the most useful format, and they can be uploaded as “Source data” files linked to a main figure or table
- Include model definition files including the full list of parameters used
- Include code used for data analysis (e.g., R, MatLab)
- Avoid stating that data files are “available upon request”

Please indicate the figures or tables for which source data files have been provided:

Figure 2🡪 Source data 1 & 2

Figure 3 🡪source data and source data for Fig 3 supplement

Figure 5 🡪 source data and source data Fig Supplement 1

Figure 6 🡪 Source data 1 and 2
